# The salivary microbiome as an indicator of carcinogenesis in patients with oropharyngeal squamous cell carcinoma: A pilot study

**DOI:** 10.1038/s41598-017-06361-2

**Published:** 2017-07-19

**Authors:** Axel Wolf, Christine Moissl-Eichinger, Alexandra Perras, Kaisa Koskinen, Peter V. Tomazic, Dietmar Thurnher

**Affiliations:** 1 Department of Otorhinolaryngology, Medical University of Graz, Auenbruggerplatz 26, 8036 Graz, Austria; 2 Department of Internal Medicine, Medical University of Graz, Auenbruggerplatz 15, 8036 Graz, Austria; 3 BioTechMed, Mozartgasse 12/II, 8010 Graz, Austria; 4 Department of Microbiology and Archaea Center, University of Regensburg, Universitätsstrasse 1, 93053 Regensburg, Germany

## Abstract

This study aimed to undertake an initial, comparative analysis of the oral salivary microbiome of patients with oral and oropharyngeal squamous cell carcinoma versus healthy controls. This project, conceived as a pilot study, included 11 patients (1 female, 10 male, mean age 61.6 yrs., SD = 8.2 yrs.) and 11 healthy controls (1 female, 10 male, mean age 46.7 yrs., SD = 15.1 yrs.). Samples of saliva were analysed by high-throughput sequencing of the 16S rRNA gene using the MiSeq platform. Sequence data revealed microbial changes that may mirror disease progression and reflect clinical preconditions such as age, alcohol consumption, tumour size, lymph node status, smoking habit, and tumour HPV-positivity. Consequently, mapping microbial changes in patients with oral and oropharyngeal squamous cell carcinomas might improve our understanding of the pathobiology of the disease, and help in the design of novel diagnostic and treatment strategies.

## Introduction

Squamous cell carcinoma (SCC) is a malignant epithelial tumour that originates from the mucosa of the upper respiratory tract. Cancers that arise from the oral cavity and pharynx comprise approximately 2.9% of newly diagnosed cases^1, 2^ with tobacco use, alcohol consumption, and infection with the human papilloma virus^3^ (HPV) serving as major risk factors^2, 4^. Diagnosis is performed by clinical, radiologic, and histopathologic examination. Currently, treatment modalities for SCC comprise surgical approaches (i.e. excision of the diseased tissue) as well as radiotherapy and/or chemo (-immuno) therapy^2^. However, despite improved therapy, the disease is frequently diagnosed at an advanced stage which leads to a relatively poor therapeutic outcome^5^. To improve outcome we need to optimise therapy, which necessitates an accurate detection of SCCs and assessment of disease severity. In this respect, informative biomarkers have become particularly important in helping to guide clinical decision-making. General examples include salivary proteomic analysis in autoimmune diseases or fecal biomarkers (e.g. calprotectin and lactoferrin) in inflammatory bowel diseases^6, 7^. A very intriguing alternative is the use of oral microbes as salivary biomarkers^8–12^. In addition, the human microbiome is, itself, now considered to be a potential biomarker given that microbial ecology can mirror or even provoke substantial changes to physiology^9, 13^.

The surface of the human body harbours trillions of microorganisms with numerous functions and capabilities that are closely aligned with human health and well being. Further, dysbiosis (i.e. an imbalance of the human microbial community) has been linked to inflammatory bowel disease, diabetes, obesity, and mental illnesses such as depression^14–16^.

Certain cancers have been associated with an altered microbial profile, such as gastric, oral^17^, lung^18, 19^, pancreatic^20^ and colonic malignancies^21^. Further, microbial composition not only reflects carcinogenesis, but can also impact anticancer immunotherapy via indirect CTLA-4 blockage^22–24^. Causal roles for microorganisms in cancer have also been described (e.g. *H. pylori* (gastric cancer)^25^ or *Salmonella typhi* (gallbladder cancer)^26^. Inflammation in response to microbes or their products (endotoxins or enzymes), as well as direct DNA damage (provoked by reactive nitrogen species, reactive lipids, metabolites, or metalloproteases), can modulate the tumour microenvironment and influence cell proliferation. These responses constitute injurious stimuli that can then drive tumourigenesis^27–29^.

The human oral microbiome comprises more than 1,000 different microbes, inclusive of bacteria, archaea, viruses, and eukaryotes^30^. Streptococci are the dominant bacteria, usually accompanied by *Veillonella*, *Gemella, Rothia*, *Fusobacterium*, and *Neisseria* species^31, 32^. Ordinarily, a well-balanced oral microbiome co-exists with its human host^33^. However, in cases of dysbiosis (i.e. induced by *Porphyromonas gingivalis* and *Fusobacterium nucleatum*) pathology can arise, such as periodontal disease or even cancer. In particular, *Porphyromonas* is increased at the surface of OSCCs, and is also associated with a disrupted immune response^34^.

Pathogenic microbial processes provide potential targets for cancer prevention, diagnosis, and treatment. For example, restoring eubiosis in chronic disease states may ameliorate carcinogenic effects^35^. To exploit these possibilities we need to be able to accurately catalogue microbial ecology. High- throughput sequencing of the microbial 16S rRNA gene allows a broad assessment to be made of the microbial community. In particular, microbiome-based diagnostics using saliva have received substantial interest given the ease with which these samples can be obtained, together with the wide-ranging insight that the salivary microbiome can provide given its proximity (and interaction) with the intestinal equivalent^2, 8, 36^.

Based on recent studies that described an altered microbiome in tumour and non-tumour tissues of patients with oral squamous cell carcinoma, it was concluded that the most prevalent or unique bacterial species/phylotypes present in tumour tissues may themselves be associated with OSCC^37–39^. Based on these data, we felt that further analyses of these microbial fluctuations were warranted.

Our hypothesis is that the microbial composition of saliva differs significantly between patients with oral/oropharyngeal SCC and healthy controls. Furthermore, we hypothesise that the salivary microbiome (including its functions) can reflect disease status and stage, and indicate general ongoing mechanistic processes. Therefore, interrogation of salivary microbial biomarkers could prove to be informative with respect to the pathobiology and carcinogenesis of SCC, disease detection (at an early stage), and potential therapeutic intervention^40^.

## Results

### Profiles of bacterial communities derived from saliva samples retrieved from patients and healthy controls

The 16S rRNA gene sequences harvested from the microbiome of saliva samples were sequenced using the MiSeq platform. In total, almost 790,000 raw reads were retrieved, resulting in approximately 340,000 reads following quality filtering and paired-end merging. In excess of 5,800 bacterial operational taxonomic units (OTUs) were obtained, based on grouping at 97% similarity. The cleaned OTU table is given in the Supplementary Dataset 1 (chloroplast sequences removed, OTUs with 3 or less sequences removed, unassigned reads removed). Only one OTU affiliated to the archaeal domain was found (genus *Methanobrevibacter*), with this appearing marginally in relatively few samples. Rarefaction curves are provided in Supplementary Fig. S1.

Compared to healthy controls, the Shannon Diversity Index as well as the InvSimpson Index revealed a slightly higher diversity in saliva samples taken from tumour patients, although this difference was not found to be statistically significant (Supplementary Fig. S2).

Overall, 17 bacterial phyla were detected, with most OTUs affiliated with Firmicutes (48% of all OTUs), Bacteroidetes (17%), Actinobacteria (15%), and Proteobacteria (12%). Sequences belonging to other phyla were detected at marginal levels (i.e. beneath the 5% threshold for all OTUs). These marginal phyla included Fusobacteria, Spirochaetes, TM7 (Saccharibacteria), Tenericutes, Cyanobacteria, Synergistetes, SR1 (Absconditabacteria), Thermi, GN02 (Gracilibacteria), Chloroflexi, Armatimonadetes, OP3 (Omnitrophica) and Verrucomicrobia (Supplementary Fig. S3).

**Figure 1:**
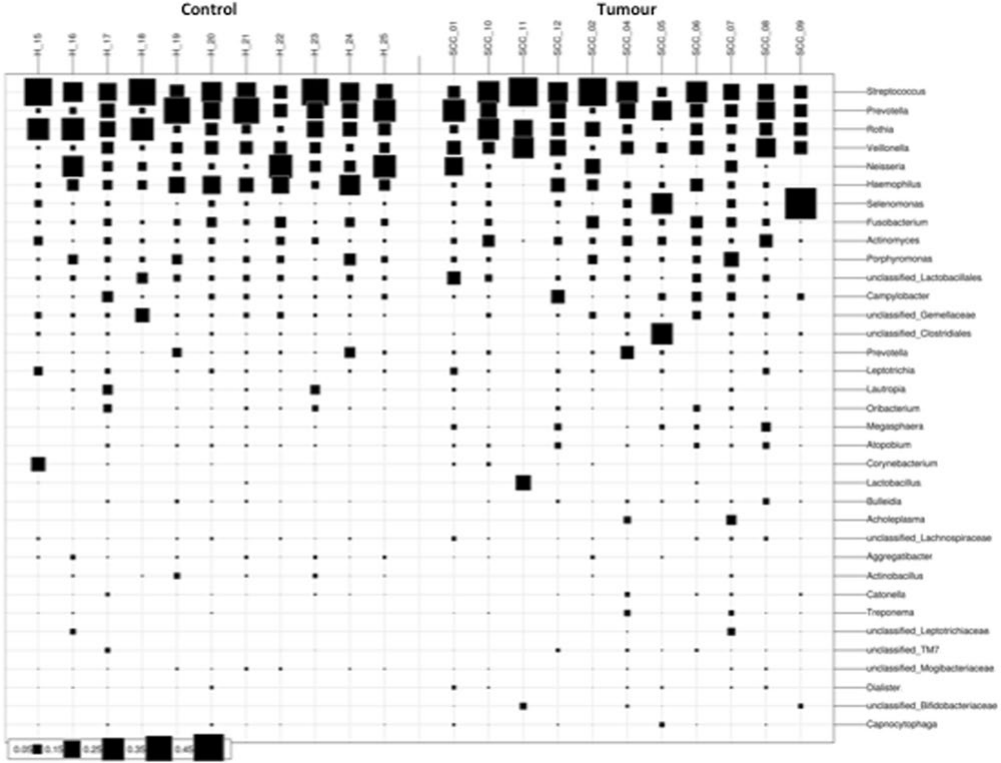
Bubbleplot of the 35 most abundant bacterial genera detected in saliva samples of healthy controls (‘H’) and cancer patients (‘SSC’).

The highest percentages of reads were assigned to the bacterial genera *Streptococcus* (21%), *Prevotella* (13%), and *Rothia* (12%; Supplementary Fig. S3). Signatures of multiple microbial species appeared to be more or less evenly distributed amongst the samples although some species (*Lactobacillus* or *Corynebacterium*; Fig. 1) appeared to be specific to certain patient samples. Due to the hetereogeneity of the tumour samples, this grouping was found to be statistically significant by Adonis analysis (p = 0.026).

**Figure 2:**
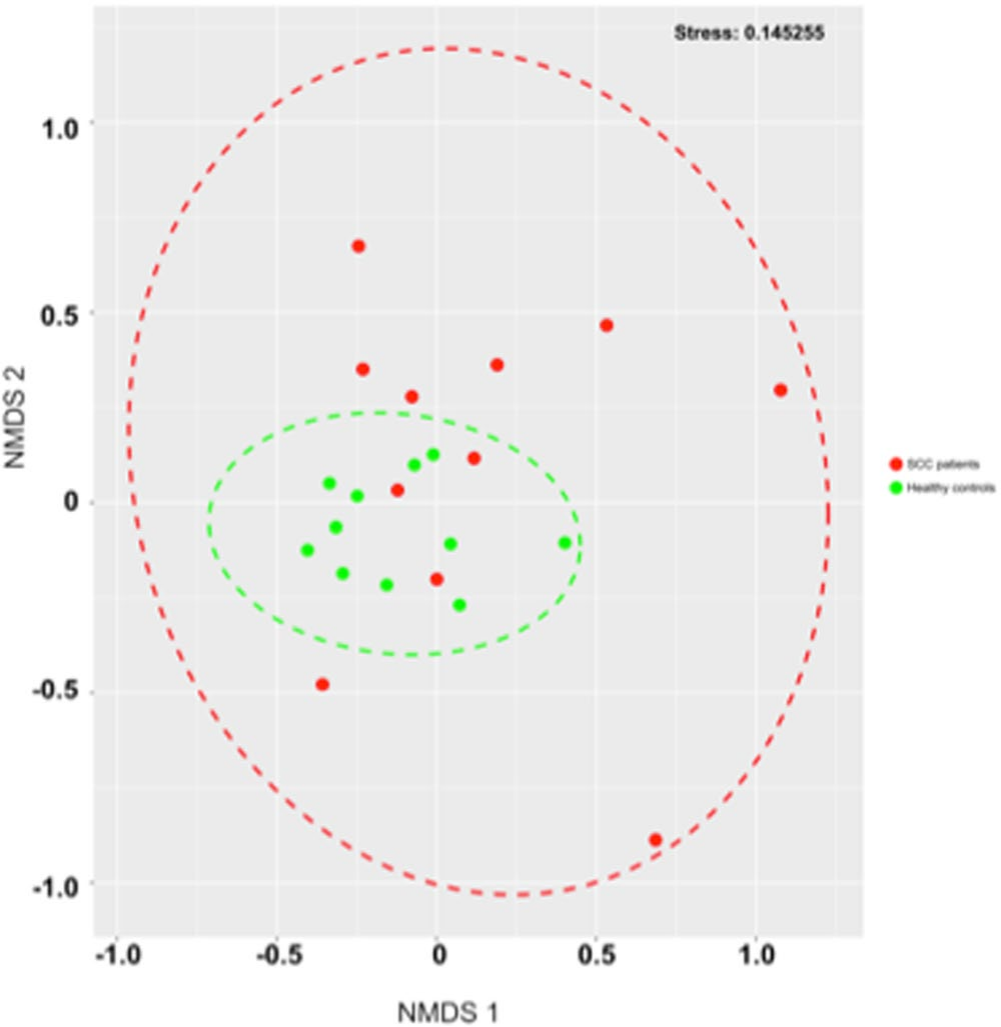
Non-metric multidimensional scaling (NMDS) plot of microbial communities, based on OTU level, derived from saliva of healthy controls (green) and SCC patients (red). Red dots within the green circle represent HPV-positive patients.

When comparing the microbial community of cancer patients vs. healthy controls at the OTU level (using non-metric multidimensional scaling, NMDS), healthy controls were present as a subcluster of the SCC patients (Fig. 2).

Several significantly different microbial taxa (*p* < 0.05) were found when comparing patient and control data. The Linear Discriminant Analysis Effect Size (LEfSE) was performed on a multi-level basis (phylum, class, order, family, genus, and species level). The retrieved, significantly different bacterial taxons are given in Supplementary Fig. 4, which depicts, besides an overview, these differences based on representative taxa (Proteobacteria, Spirochaetae, Mollicutes_NB1_n, *Stomatobaculum*, Peptostreptococcaceae and γ- Proteobacteria), and their abundance at a single sample level for healthy controls and tumour patients. The highest LDA score was retrieved from Proteobacteria as a whole taxon (highest in tumour patients; Supplementary Fig. S4).

**Figure 3:**
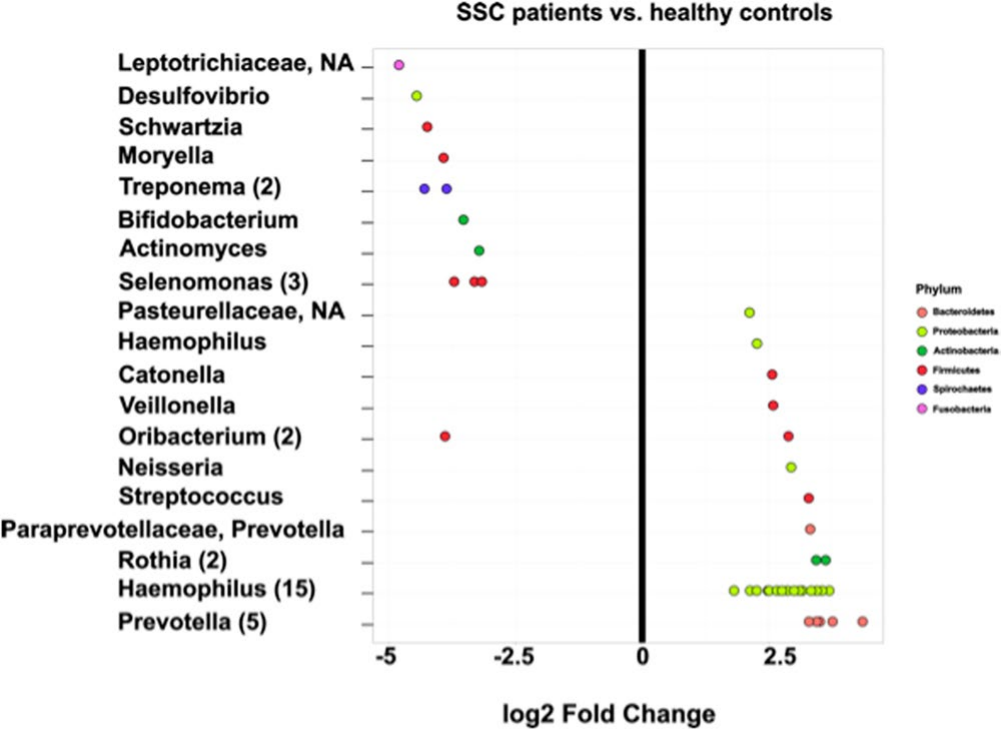
Pairwise comparison (DeSeq analysis). Differential abundant OTUs (p < 0.05) in SSC patients and healthy control counterpart samples are shown. OTUs were assigned to genus (y-axis) and phylum level (colours) and amount of respective OTUs (when >1) are shown in brackets. Negative “log2 Fold Change” values (x-axis) indicate for higher abundance in SSC patient samples and positive values indicate higher abundances in healthy controls.

A more detailed, OTU-level based, pairwise comparison between SSC patient samples and healthy controls revealed 45 differentially abundant OTUs (DESeq analysis, p < 0.05; Fig. 3). Signatures of the genera belonging to the phylum Bacteroidetes (*Prevotella*), Proteobacteria (e.g. *Haemophilus* and *Neisseria*) and Firmicutes (e.g. *Streptococcus* and *Veilonella*) were higher abundant in healthy controls, whereas signatures affiliated to *Actinomyces* (Actinobacteria), *Schwartzia* (Firmicutes), *Treponema* (Spirochaetes) and *Selenomonas* (Firmicutes) were higher abundant in SSC patients.

**Figure 4:**
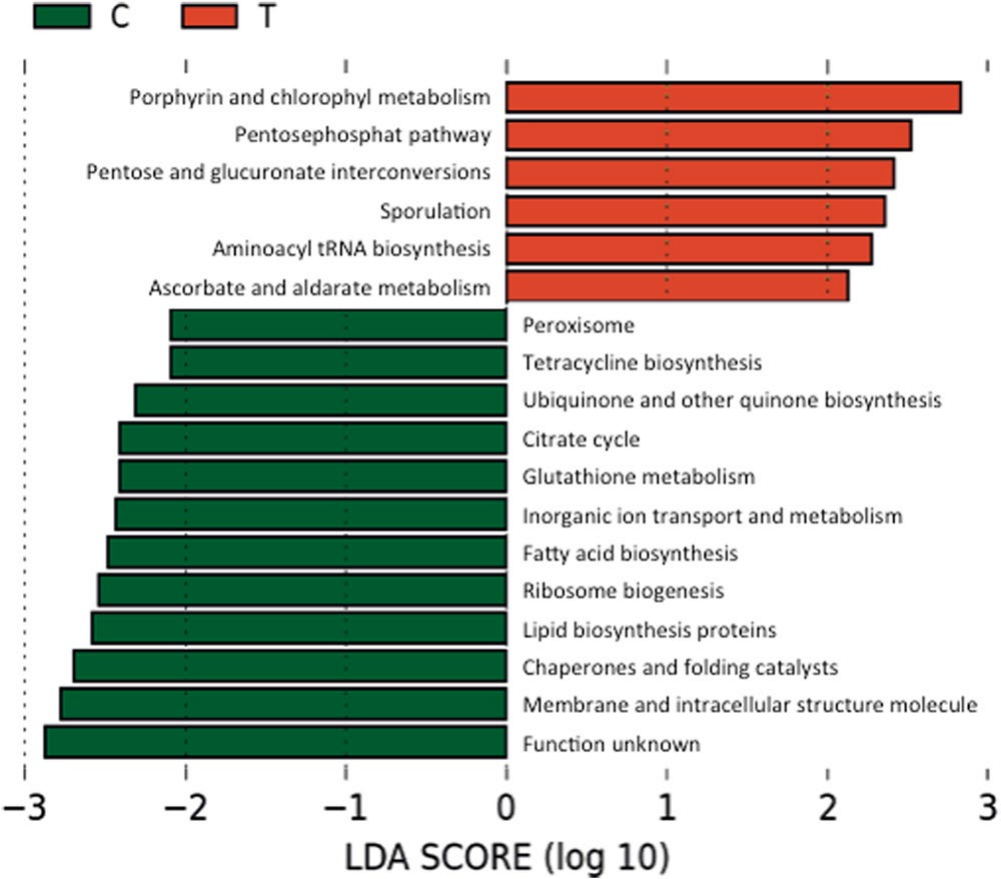
LEfSE analysis, indicating the potential presence of significantly different genomic/ functional capabilities of the microbiomes of tumour patients (red) and healthy controls (green).

From PICRUSt (Phylogenetic Investigation of Cummunities by Reconstruction of Unobserved States) and subsequent LEfSE analyses significant differences in genomic features when comparing the estimated functional capabilities of microbiomes derived from tumour patients vs. healthy controls could be inferred (Fig. 4). Signals from tumour-associated samples indicated an increased abundance of genes involved in porphyrin metabolism (co-factor and vitamin production), pentose phosphate pathway (carbohydrate metabolism), pentose and glucuronate interconversion (carbohydrate metabolism), sporulation (microbial endurance and dormancy), and ascorbate and aldarate metabolism (sugar metabolism). In comparison, controls showed a potential increased gene involvement in lipid and fatty acid biosynthesis, inorganic ion transport and metabolism, glutathione metabolism (amino acid metabolism), the citrate cycle/TCA cycle, ubiquinone and other terpenoid quinone biosynthesis pathways (co-factor and vitamin production), and tetracycline biosynthesis. Overall, these significantly different (predicted) functions indicate a stronger focus of the tumour microbiome on sugar metabolism (including biofilm matrix formation) and the stress response (spore formation), whereas healthy controls were skewed towards lipid metabolism and defence mechanisms (e.g. tetracycline biosynthesis).

**Figure 5:**
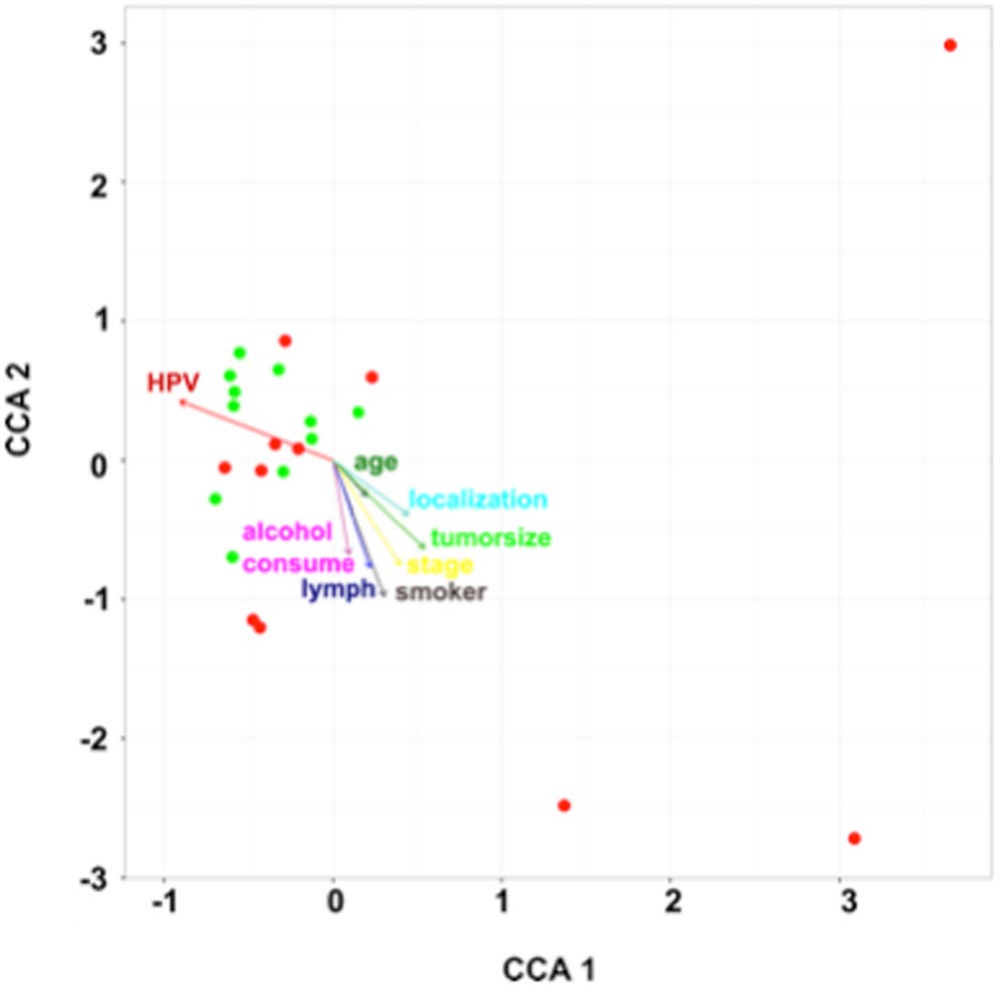
Canonical correspondence analysis (CCA) of the bacterial communities with various factors.

When mapping the microbiome, metadata were retrived for age, alcohol consumption, tumour size, lymph node status, and, smoking. These were subsequently identified (see Supplementary Fig. S5) as potential drivers in shaping a tumour-associated microbiome (Fig. 5).

However, patients diagnosed as positive with respect to HPV infection (see Table 1) demonstrated a “normal” microbiome, i.e. a microbiome composition that resembled healthy controls as demonstrated in Fig. 2, where samples from HPV-positive patients cluster with healthy controls (red dots within the green group).

**Table 1:** Descriptive data of the study population.

ID	Group	Age (years)	Sex (m = male,f = female)	Smoker	Alcohol consumption	Tumour localisation	Tumour side	Tumour size (T stage)	Lymph nodes metastasis (N stage)	Distant metastasis (M stage)	HPV	Stage of disease	Comorbidity
1	patient	54	m	yes	occasional	oropharynx	Tongue base	4b	3	0	negative	4	none
2	patient	68	m	no	never	oropharynx	Tongue base	1	2b	0	positive	4	arterial hypertension, hypercholesterolemia
3	patient	57	m	yes	daily	oral cavity	mouth floor	4a	2b	0	negative	4	Wernicke-encephalopathy
4	patient	54	m	no	occasional	oropharynx	soft palate	1	0	0	negative	1	arterial hypertension
5	patient	64	m	yes	occasional	oral cavity	mouth floor	2	2c	0	negative	4	arterial hypertension
6	patient	61	m	yes	daily	oral cavity	mouth floor	2	2c	0	negative	4	frequent alcohol consumption
7	patient	55	f	yes	occasional	oral cavity	mouth floor	1	0	0	negative	1	none
8	patient	67	m	yes	daily	oropharynx	tonsil	4a	2c	0	negative	4	none
9	patient	64	m	no	occasional	oropharynx	tonsil	4a	2b	0	positive	4	none
10	patient	53	m	yes	daily	oropharynx	Tongue base	4a	2b	0	negative	4	none
11	patient	80	m	no	occasional	oropharynx	Tongue base	1	1	0	positive	3	none
12	control	66	m	no	occasional	n.a.	n.a.	n.a.	n.a.	n.a.	n.a.	n.a.	none
13	control	59	m	no	none	n.a.	n.a.	n.a.	n.a.	n.a.	n.a.	n.a.	none
14	control	68	m	no	none	n.a.	n.a.	n.a.	n.a.	n.a.	n.a.	n.a.	none
15	control	55	f	yes	occasional	n.a.	n.a.	n.a.	n.a.	n.a.	n.a.	n.a.	none
16	control	53	m	no	occasional	n.a.	n.a.	n.a.	n.a.	n.a.	n.a.	n.a.	none
17	control	43	m	no	occasional	n.a.	n.a.	n.a.	n.a.	n.a.	n.a.	n.a.	none
18	control	29	m	no	occasional	n.a.	n.a.	n.a.	n.a.	n.a.	n.a.	n.a.	none
19	control	60	m	no	occasional	n.a.	n.a.	n.a.	n.a.	n.a.	n.a.	n.a.	none
20	control	31	m	no	occasional	n.a.	n.a.	n.a.	n.a.	n.a.	n.a.	n.a.	none
21	control	31	m	no	occasional	n.a.	n.a.	n.a.	n.a.	n.a.	n.a.	n.a.	none
22	control	31	m	no	occasional	n.a.	n.a.	n.a.	n.a.	n.a.	n.a.	n.a.	none
**Total**													
Patients	n = 11	mean patients 61.6(8.2)	m = 10,f = 1	Smokers = 7, non-smokers = 4	never = 1; occasional = 6; daily = 4	oropharynx = 7, oral cavity = 4					negative = 9, positive = 2		
Control	n = 11	mean control 47.7(15.2)	m = 10, f = 1	Smokers = 1, non-smokers = 10	never = 2; occasional = 9								
Overall	n = 22	mean overall 54.7(13.8)	m = 20, f = 2	Smokers = 8, non-smokers = 14	never = 3; occasional = 15; daily = 4								

Information about tumour size, lymph node metastasis, distant metastasis and stage of diseases is provided according to TNM classification. Standard deviations are set in parenthesis.

## Discussion

This study aimed to undertake a comparative analysis of oral microbial communities using the saliva of patients with oral and oropharyngeal squamous cell carcinoma versus healthy controls. In this pilot study we found initial evidence that differences in microbial abundance and diversity might inform disease status in SCC patients. These findings might influence our understanding of disease pathogenesis, and could be used as the basis for further studies on novel diagnostic and treatment strategies.

In earlier studies of the microbial ecology of saliva, culture based methods were primarily used and the majority of microbial community remained hidden^41–47^. However, advances in DNA sequencing have made it possible to undertake in-depth analyses of microbes that are not amenable to culture using high-throughput sequencing of the microbial 16 S rRNA gene^48, 49^. A deeper knowledge into the contribution of the microbiota to cancer proliferation may help to develop novel treatments and early recognition. The potential of using the microbiome as diagnostic biomarkers has been recognized before, as several, recent studies report on an altered salivar microbial composition in cancer patients when compared to healthy counterparts.

Previously, Guerrero-Preston *et al*. characterised saliva microbiota in the oropharynx and cavity of squamous cell carcinoma patients, comparing these to healthy controls. Using the 16 S rRNA V3-V5 marker gene approach, saliva was examined in patients before and after surgical tumour resection. Head and neck squamous cell carcinoma (HNSCC) patients manifested a significant loss in the richness and diversity of microbiota species, with specific OTUs used to discriminate patients from controls. Further, longitudinal analyses revealed a reduction in the alpha-diversity measure following surgery, which then increased in patients with recurrent disease^50^.

Wang *et al*. performed a matched-pair analysis of individual tumour and paired-normal tissue and observed significant differences in the relative abundance of the genus *Actinomyces*. These differences were more pronounced among patients with progressed T-stages of disease. It was suggested that potential unknown mechanisms relevant to carcinogenesis were associated with the altered oral microbiome^38^.

Mager *et al*. investigated microbial abundance in a case-control study. Although they evaluated samples for their microbial content using checkerboard DNA-DNA hybridization of only 40 bacteria, they suggested that the abundance of certain microbial species (i.e. *Capnocytophaga*, *Prevotella*, and *Streptococcus*) could be used as diagnostic markers for oral squamous cell carcinoma^17^.

Agreeing with our findings, a high prevalence of Firmicutes was observed earlier in the oral microbiome of SCC patients and in healthy subjects^37, 41, 50–52^. Schmidt *et al*. and Wang *et al*. describe that Firmicutes (especially *Streptococcus*) and Actinobacteria (especially *Rothia*) are significantly decreased in cancer patients when comparing tumour sites with contralateral non-tumour samples from the same patient. Thus, they suggested that oral lesions may be associated with shifts in the oral microbiome that contribute to cancer development^38, 53^. However, in contrast to our investigation, both groups profiled the microbiome of cancer tissue and anatomically matched contralateral normal tissue, but did not perform a case-control study that included healthy subjects.

Nagy *et al*. analysed oral biofilms in 21 OSCC patients, comparing tumour and non-tumour sites. An increased colonisation with *Prevotella*, *Fusobacteria, Porphyromonas, Actinomyces, Clostridium, Haemophilus*, Enterobacteriaceae, and *Streptococcus* spp. and *Veillonella* ^42^ was detected at the tumour sites. In congruence, our cohort revealed a significant decline of signatures affiliated with *Veillonella* (Firmicutes), *Prevotella*, and *Streptococcus* in our patient group, whereas an increase of *Actinomyces* signatures in SSC patients was observed. Interestingly, in particular the relative abundance of *Actinomyces* and parent taxa were detected to be significantly depleted also in HNSCC patients, however, there is no hint on if and how the microbial shifts influence cancer profileration^38^.

Another study, on the other hand, evidenced *Fusobacterium nucleatum* to possible mediate tumour profileration by activating several chemokines^54^. Microbial shifts may be provoked by altered microenvironments that disturb the normal salivary microbiome and, consequently, lead to a different microbial composition. Several bacterial mechanisms could affect carcinogenesis in SCC. For example, streptococci produce short chain organic acids that lower the local pH. Thus, they may contribute to the production of an acidic and hypoxic tumour environment^55, 56^.

In general, chronic infections or direct alteration of the microbial metabolome, including that driven by endotoxins, enzymes, and metabolic byproducts can influence carcinogenesis. Microbes and their products activate human cells (e.g. neutrophils, macrophages, monocytes, lymphocytes, fibroblasts and epithelial cells) and can result in the generation of reactive species including nitrogen species, reactive lipids and metabolites, and metal proteases^57^. These substances can induce mutations of tumour suppressor genes and proto-oncogenes, as well as alter pathways that control cell proliferation and/or survival^37, 58, 59^. This dysregulation influences cell growth, invasion, tumour suppression, immunity, and ultimately survival^60^. Thus, these mediators are critical for the development and/or growth and/or progression of disease. With these mechanisms in mind, it is plausible to assume that bacterial ecology influences pathogenesis, although whether this is a causal relationship, or simply a microbial reaction to an altered microenvironment, cannot be established with certainty.

According to our results, very recent studies identified shifts within the microbiome of the oral cavity associated with cigarette smoking^61^. The link between bacterial ecology, smoking, and carcinogenesis are ill-defined, bacteria might play a role in the increased activation of carcinogenic nitrosamines^62, 63^. Charlson *et al*. described an analysis of upper airway colonisation in smokers and non-smokers in a relatively large number of participants (n = 62). In multiple tests of a longitudinal study, these authors reported stable differences in the oral microbiome of smokers vs. non-smokers^64^. According to the literature, and the current results, smoking appears to drive microbial changes to saliva that may predispose to respiratory tract disease^64^.

In addition, alcohol consumption is considered as a potential driving factor for the composition of the salivary microbiome. Microbial changes of the gastrointestinal tract associated with alcohol consumption are described in literature^65^. Although alcohol itself is not known to be carcinogenic, acetaldehyde (its first metabolite) can cause genomic damage that might influence carcinogenesis^66^.

HPV infection, in particular HPV 16, is, epidemiologically, an increasingly relevant risk factor for SCC of the oropharynx. Recently, Guerrero-Preston *et al*. investigated microbial ecology in patients diagnosed with head and neck SCC vs. healthy controls. They described a significant abundance of the genus Gemellaceacae and *Leuconostoc* in HPV+ HNSCC patients compared to HPV− HNSCC patients^50^. Due to the low number of analysed patients, we were unable to retrieve specific markers for HPV+ patients in our study. However, we could identify a potential (controversial) link between HPV+ tumours and the salivary microbiome when comparing age, smoking, alcohol consumption, lymph node status, and tumour size.

These findings might be explained by the fact that HPV+ tumour patients clearly differ from HPV− tumour patients in terms of their clinical characteristics. For example, HPV+ OSCC cases are prevalent in younger patients with less tobacco and alcohol consumption. In accordance with the literature, HPV+ patients in our cohort are non-smokers, with a limited alcohol intake, and with primary tumours in the base of the tongue and tonsils.

The main limitation of this pilot study is its limited sample size and the heterogeneity of the patient and control groups in terms of their demographics and clinical parameters. The factors that we identified as influencing microbial abundance (smoking, alcohol consumption, disease stage, HPV-status, and medical history) are also potential confounders. Further studies should include larger cohorts in order to more fully explore the impact of these factors on our findings. Additionally, metagenomics studies should be envisaged, which could give deeper insights into the functional capacity of the microbial community and extend the information retrieved by PICRUSt estimation.

The Salivette® Cortisol kit was a user-friendly method with which to collect saliva from both the oral cavity and oropharynx. However, specific differences between the oral and oropharyngeal salvia could not be evaluated using this sampling procedure.

Overall, the results of this study have shown differences of the salivary microbiome between oral and oropharyngeal SCC patients and healthy controls. Salivary microbial biomarkers are a promising information source with respect to SCC carcinogenesis, disease detection, and potential therapeutic interventions. Microbial changes may mirror disease status and clinical pre-conditions such as age, alcohol consumption, tumour size, lymph node status, smoking habit, and HPV infection. We anticipate that further studies will expand on these data, and findings in patients and controls in terms of clinical conditions such as HPV status.

## Materials and Methods

Participants were recruited from the Department of Otorhinolaryngology, University hospital of Graz. The study was approved by the Ethics Committee of the Medical University of Graz (1325/2016) and conducted according to the guidelines of the Declaration of Helsinki on biomedical research involving human subjects. All participants provided written informed consent.

### Study subjects

The study was conceived as a pilot study and included 11 patients (1 female, 10 male, mean age 61.6 yrs. (SD = 8.2 yrs.), and 11 healthy controls (1 female, 10 male, mean age 46.7 yrs. (SD = 15.1 yrs.). Patients diagnosed with OSCC and SCC of the oral cavity were included. Immunohistochemical detection of the p16 protein was used as surrogate marker for HPV status. A summary of the clinical parameters of the participants is shown in Table 1.

The participants did not take any local or systemic antibiotics for at least four weeks before their inclusion in the study. Patients and controls had neither undergone long-term antibiotic use (as per their medical histories), nor had they been vaccinated in the six months prior to study inclusion.

### Sampling procedure

Saliva was collected from the mouths of patients after their primary diagnosis of SCC, but prior to any specific treatment including surgery, radiotherapy, or chemo (immuno-) therapy. Samples were collected using the Salivette® Cortisol saliva detection kit (Sarstedt, Newton, NC, USA), which contains a small polyester swab for saliva absorption and a conical tube for centrifugation and recovery of the saliva. Subjects were asked to chew on the polyester swab for about 60 seconds to stimulate salivation before placing the swab, with the absorbed saliva, back into the tube. A 5 minute centrifugation at 3,000 rpm was used to collect a clear saliva sample that was then (immediately) stored at −70 °C until use.

### DNA extraction, amplicon-production, high-throughput sequencing, and sequence processing

Microbial DNA was extracted using the Magna Lyser instrument and the MagNA pure LC DNA Isolation Kit III (Roche Diagnostics, Mannheim, Germany), according to manufacturer’s instructions. DNA was quantified using Qubit (Thermo Fisher Scientific) with 0.5–15 ng of the extracted DNA used for subsequent amplification of the microbial 16 S rRNA gene pool (using primers 515 F and 806R^67^). The amplification protocol has been described elsewhere^68^. Sequencing was performed using Illumina MiSeq, a service provided by the Center for Medical Research (ZMF) at the Medical University Graz.

Raw reads were processed via QIIME following standard operating procedures (sequence length >200 bp). In brief, reads were checked for the presence of chimeras (usearch 61; reference database: GreenGenes 13_8) and then grouped into OTUs at the 97% level. QIIME was run on an internal Galaxy set-up (ZMF, Medical University Graz)^69^. Read classification (open reference picking) was performed using the recent GreenGenes database 13_8^70^.

Unassigned sequences and chloroplast signatures were removed, as well as OTUs represented by 3 or less sequences. The OTU table (Supplementary Dataset 1) was processed in Calypso^71^. The dataset was normalised via TSS (total sum normalisation). Minimal reads per samples were observed in sample SCC_09 (98,694 reads), whereas maximal reads per sample were retrieved from SSC_12 (221,252 reads). Calypso was used to calculate Adonis (OTU level), Inverse Simpson Index, Shannon Index (OTU level), bar chart (genus level), bubble plot (genus level), and rarefraction curves (OTU level).

Output data was also processed using Lefse^72^, MaAsLin^73^ and R studio (v.3.3.0). NMDS and CCA plots were generated by using the R packages vegan and phyloseq^74^, and differential genera abundance was analysed by using a negative Binomial method implemented in the package DESeq275 as recommended by McMurdie and Holmes^75^. Phylogenetic Investigation of Communities by Reconstruction of Unobserved States (PICRUSt)^76^ was used for functional assessment. Negative controls (extraction blanks) were processed in parallel but did not reveal substantial sequence reads. Raw sequence reads were submitted to GenBank and are publicly available (PRJEB18476).

### Ethics

This study was approved by the ethics committee of the Medical University Graz (EK-Nr. 1325/2015). Experimental protocols were approved by the ethics committee and the Department of Otorhinolaryngology of the Medical University of Graz.

## Electronic supplementary material

Supplementary table

Supplementary figures
