# Supplementary material for: The salivary microbiome as an indicator of carcinogenesis in patients with oropharyngeal squamous cell carcinoma: A pilot study
Source: Sci Rep. 2017 Jul 19;7:5867. doi: 10.1038/s41598-017-06361-2 (PMC5517471; doi:10.1038/s41598-017-06361-2)

The salivary microbiome as an indicator of carcinogenesis in patients with oropharyngeal squamous cell carcinoma: A pilot study

Axel Wolf, MD[1], Christine Moissl-Eichinger[2,3] *, Alexandra Perras[2,4], Kaisa Koskinen[2,3], Peter V. Tomazic, MD[1] and Dietmar Thurnher, MD[1] *

1 Department of Otorhinolaryngology, Medical University of Graz, Auenbruggerplatz 26, 8036 Graz, Austria

2 Department of Internal Medicine, Medical University of Graz, Auenbruggerplatz 15, 8036 Graz, Austria

3 BioTechMed, Krenngasse 37, 8010 Graz, Austria

4 Department of Microbiology and Archaea Center, University of Regensburg, Universitätsstraße 1, 90343 Regensburg, Germany

Supplementary Figure S1: Rarefaction analysis on OTU level; richness is displayed against sampled reads. Patient samples ("Tumour"): dots and controls: triangles.

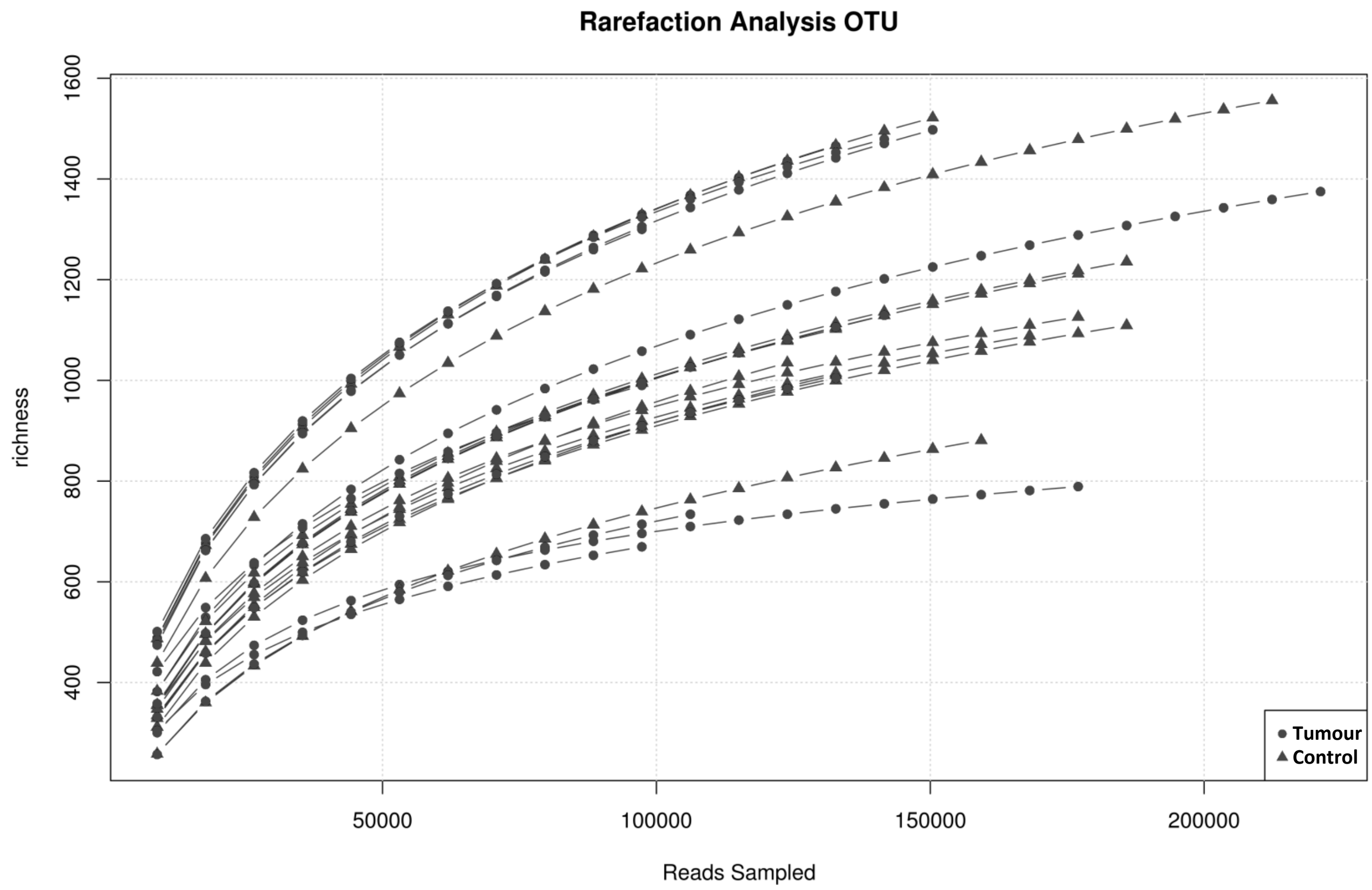

Supplementary Figure S2: Diversity Indices revealed a slightly higher diversity in tumour patients compared to healthy controls (Left diagraph: InvSimpson Index, p=0.154, Right diagraph: Shannon Index, p=0.133).

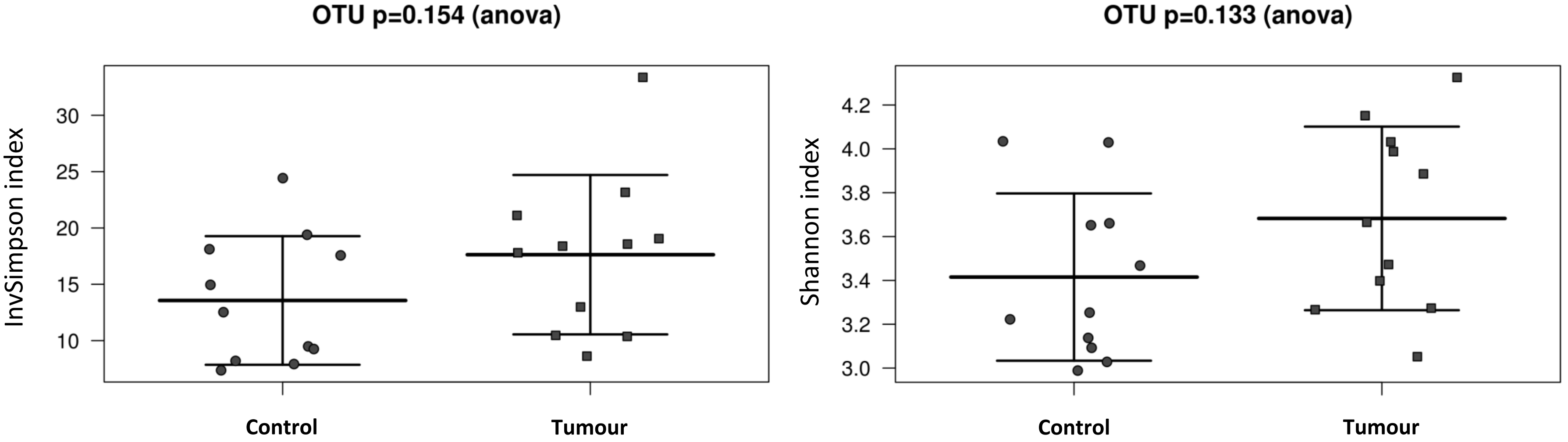

Supplementary Figure S3: Barchart, depicting the 35 most abundant microbial genera detected in the healthy controls ('H') and tumour patients ('SSC').

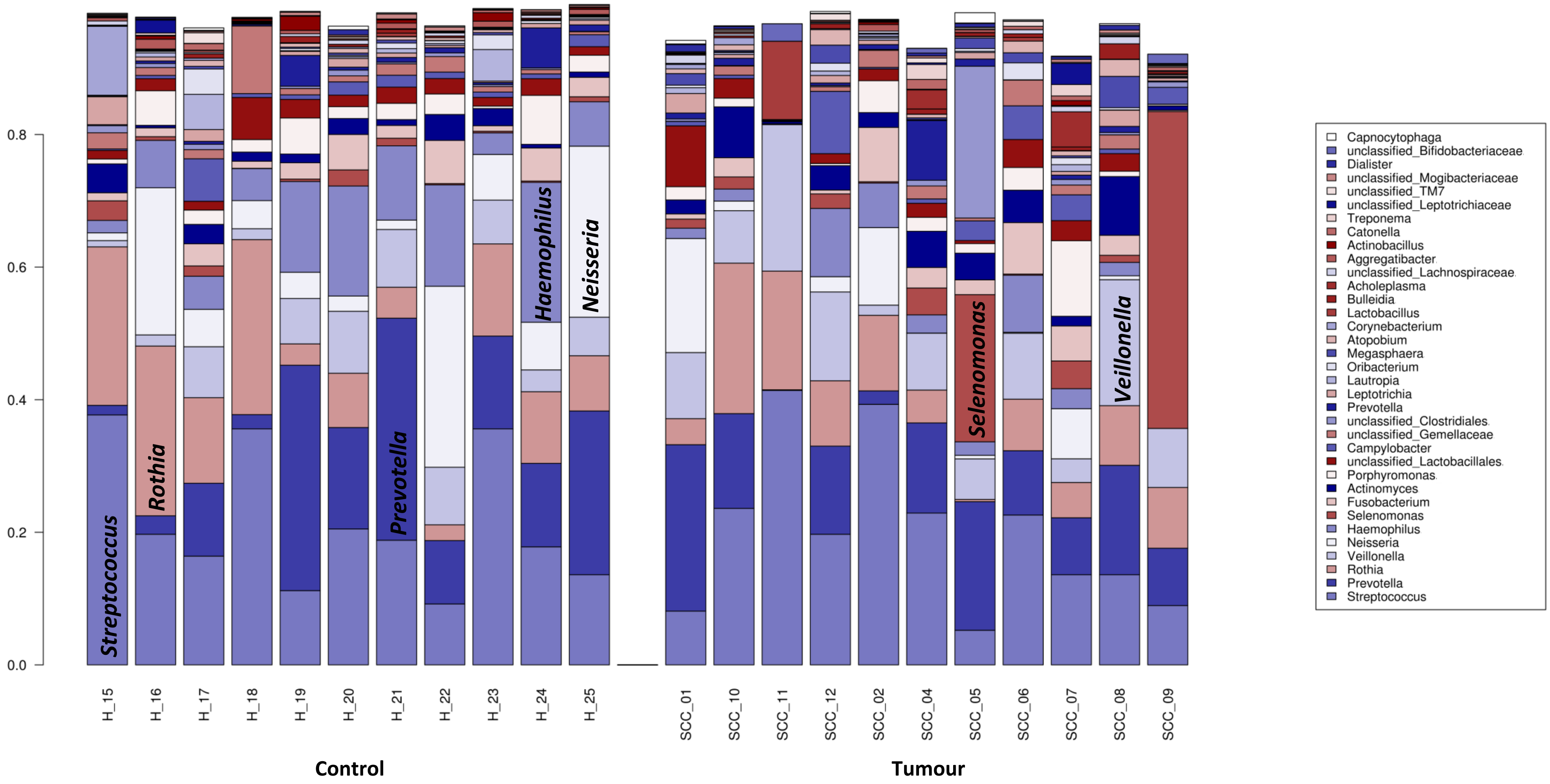

Supplementary Figure S4: Overview on microbial taxa found to be significantly associated with tumours (red, “T”) and healthy controls (green, “C”; Lefse analysis; left panel). Microbial taxa belonging to the same phylum were colored similary. Examples of microbial taxa, whose signatures were found to be significantly different between the healthy control group and tumour patients are given in the right panel. Y-axis reflects the relative abundance of microbial signatures in the dataset.

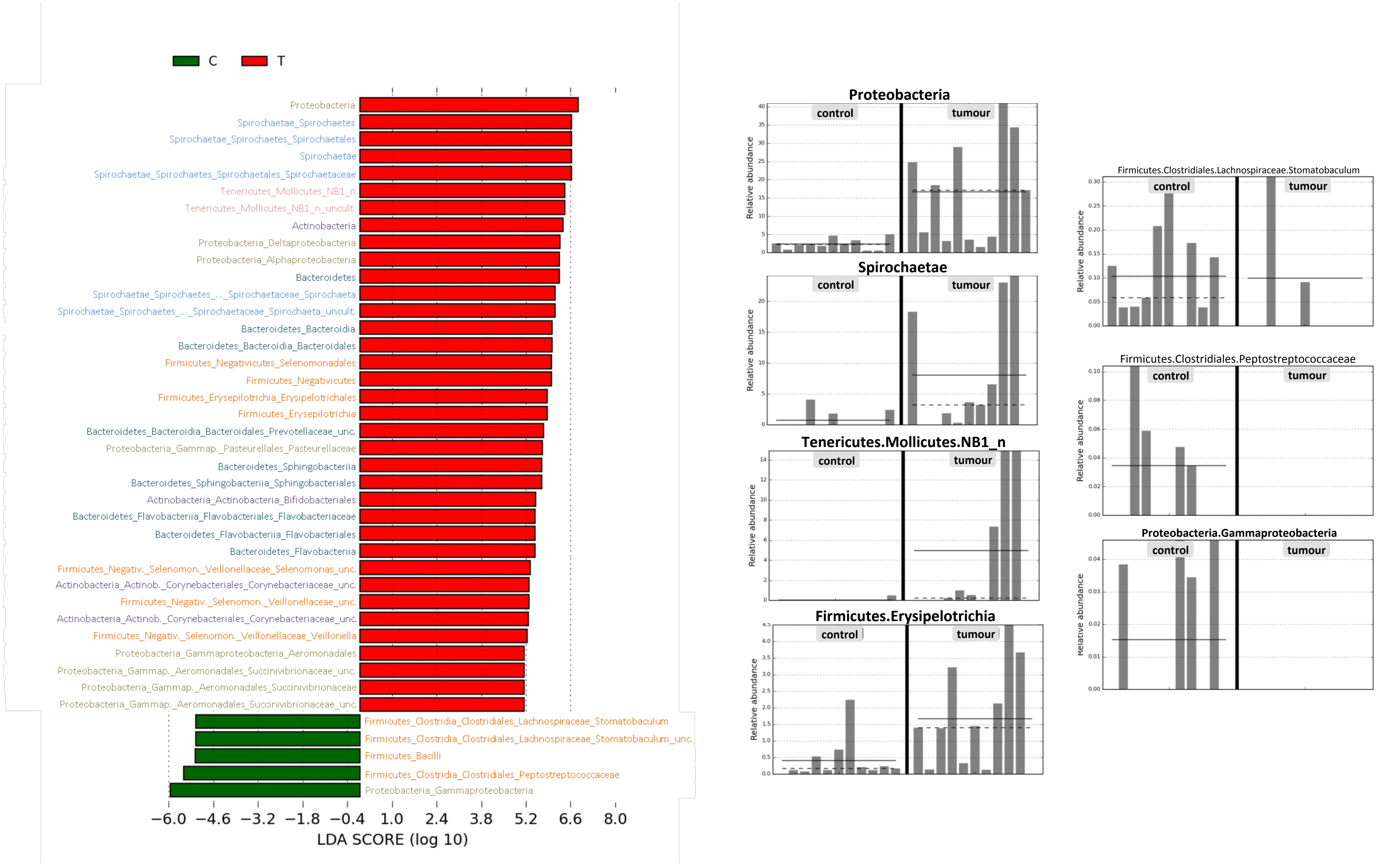

Supplementary Figure S5: Non-metric multidimensional scaling (NMDS) ordination of microbial community composition from healthy (green) and tumour patients (red) . The compositional variation is represented with Bray-Curtis distance matrix based on the abundance of OTUs. The gradients associated with ordination are tumour stage, number of lymph nodes,tumour size, localization, HPV, alcohol consume, smoke habits and age. The colors of the lines represent the respective gradient.

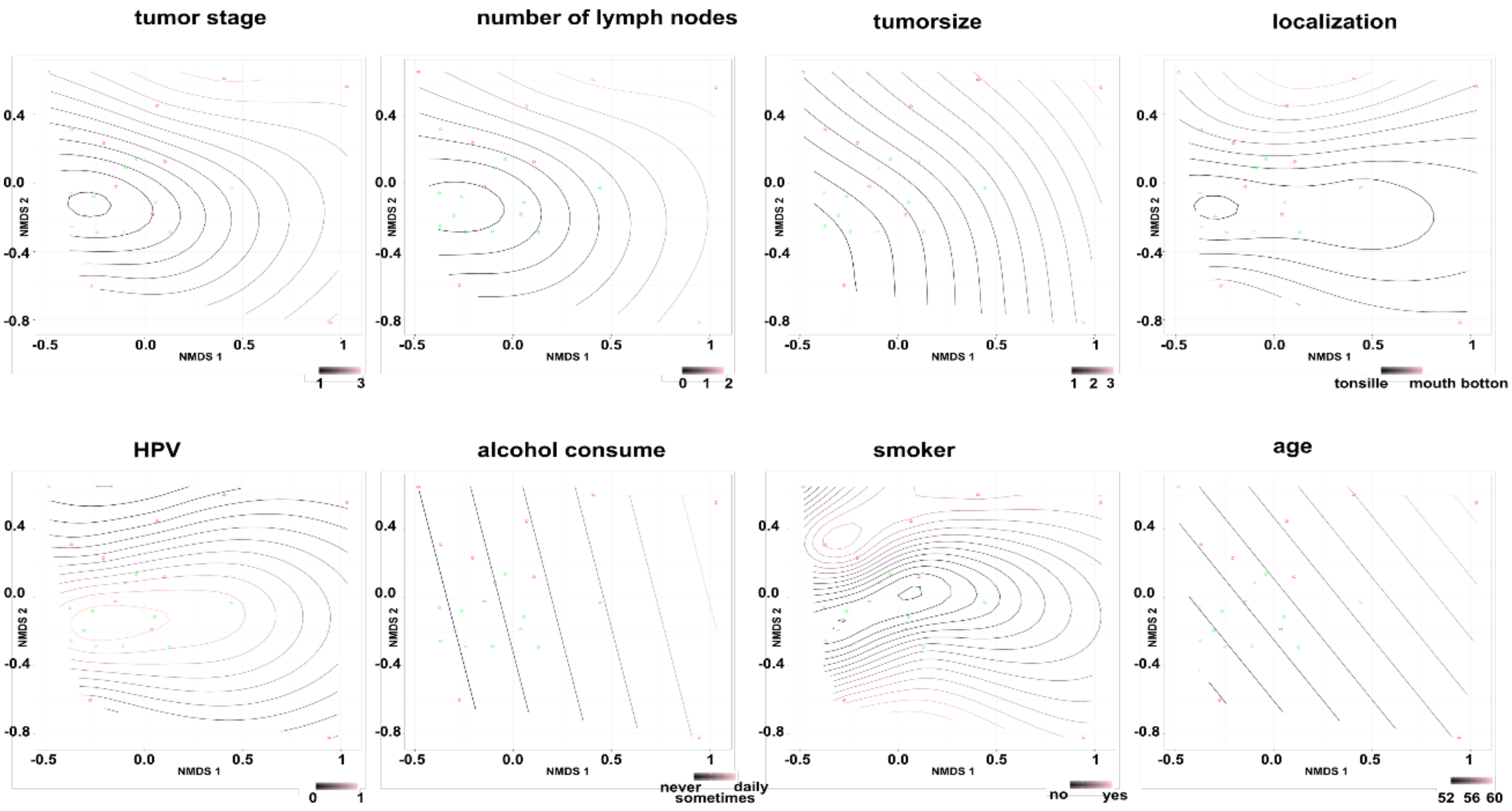

Supplement: Supplementary file 2 — Supplementary figures [file 41598_2017_6361_MOESM2_ESM.pdf]
